# Texture preference task: a rapid and training-independent behavioural assay to evaluate somatosensory perception in freely moving mice

**DOI:** 10.1016/j.mex.2026.103843

**Published:** 2026-02-21

**Authors:** Jessika Royea, Ismaël Djerourou, Behiye Sanliturk, Catherine Albert, Matthieu P. Vanni

**Affiliations:** a École d’Optométrie, Université de Montréal, Pavillon 3744 rue Jean-Brillant, Montréal, QC, Canada; b Centre Interdisciplinaire de Recherche sur le Cerveau et l’Apprentissage (CIRCA), Université de Montréal, Canada; c Department of Cellular and Molecular Medicine, University of Ottawa, ON, Canada

**Keywords:** Texture preference, Somatosensory behaviour, Open-field test, Stroke, Photothrombosis, Tactile perception, Environmental preference

## Abstract

Somatosensory perception, mediated though the whiskers and paws, plays a critical role in how rodents explore and interact with their environment. Whereas strokes are associated with devastating motor deficits, they also frequently disrupt tactile processing, yet most commonly used behavioural assays emphasize gross motor coordination rather than spontaneously driven sensory behaviour. To address this gap, we developed a simple open-field texture preference assay for mice. The task leverages innate exploratory behaviour, requires no training, and provides a rapid automated measure of tactile preference before and after cortical stroke. Here, we offer a reproducible and accessible approach for assessing post-stroke somatosensory deficits.

Essential highlights

Provides a non-invasive and training-free assay of tactile preference in freely moving mice.

Enables sensitive detection of post-stroke sensory impairments that complement standard motor tests.

Adaptable for diverse experimental contexts, including recovery studies and therapeutic interventions.

Overall, this protocol offers an efficient, low-cost addition to behavioural testing batteries in preclinical stroke research. Beyond stroke, the task may be broadly applicable to studies of tactile processing, sensory preference, and environmental enrichment in freely moving mice.

## Graphical abstract

**Figure:**
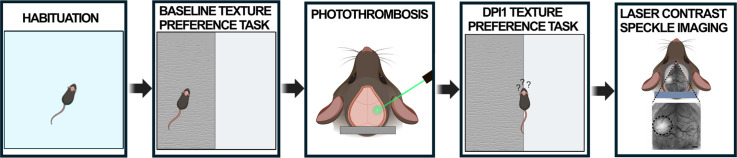

Specifications table

**Table:** 

Subject area	Neuroscience
**More specific subject area**	Somatosensory Behavioural Paradigm
**Name of your protocol**	Texture Preference Task
**Reagents/tools**	• WT Mice (C57BL/6, Charles River) • 45 Length x 45 Width x 40 Height cm open-field arena • Sandpaper, grade 36 grit (SKU: 1000,719,693, Diablo) • Arducam 1080P USB Camera (SKU: B0506, Arducam) • Red light • 70 % Ethanol • Isoflurane (Fresenius Kabi Canada Ltd) • Oxygen • Heating pad and monitoring system (Harvard apparatus) • Carprofen/Rimadyl (Best Buy Medical Supplies Inc.) • Eye lubricant (Systane) • Providone-iodine (TEVA Canada) • Lidocaine (Hikma Canada) • Cyanoacrylate (Vetbond) • Titanium head bar (Labeo Technologies Inc.) • Rose Bengal (198,250–5 G, Sigma Aldrich) • Galvano optical imaging systems (Labeo Technologies Inc.) • LightTrack OiS200 system (Labeo Technologies Inc.) • MATLAB (Mathworks)
**Experimental design**	Mice are habituated to a 45 × 45 × 40 cm open-field arena for 5 min of free exploration in the dark. Following habituation, the arena is subdivided into smooth (white board paper) and course (sandpaper) halves. Baseline texture preference is recorded during 3 min of free exploration under red light. Photothrombrotic stroke is induced and confirmed with laser speckle contrast imaging. At 1 and 3 days post-injury, mice were re-tested to assess changes in tactile preference compared to sham controls (Figure 1).
**Trial registration**	N/A
**Ethics**	All experiments conducted in this study were subjected to ethical approval from the Université de Montréal Comité de Déontologie de l’expérimentation sur les animaux (CDEA) and adhered to the Canadian Council on Animal Care guidelines. Male and female mice were both used to validate this protocol and no influence of sex was observed.
**Value of the Protocol**	• Sensitive measure of somatosensory function that captures spontaneous tactile preference, complementing standard motor assays that do not directly assess sensory-driven behaviour. • Practical and accessible task that requires minimal training, low-cost materials, and short testing sessions, making it easily adaptable across laboratories for studying sensory processing, recovery, and intervention efficacy. • Stroke relevance that provides a simple and robust readout of post-stroke tactile deficits, allowing for the detection of subtle impairments that may be overlooked by gross motor tests.

## Background

Tactile sensation is fundamental to how animals engage with their environment, guiding exploration, forging, and habitat preference [[1], [2], [3]]. In mice, somatosensory perception through the whiskers and paws plays a critical role in how they interact with their environment and plays an essential role in navigation and their affective states [[1], [2], [3]]. Despite this, few studies have examined how differences in surface texture could influence behaviour in freely moving mice [4,5].

**Fig. 1:**
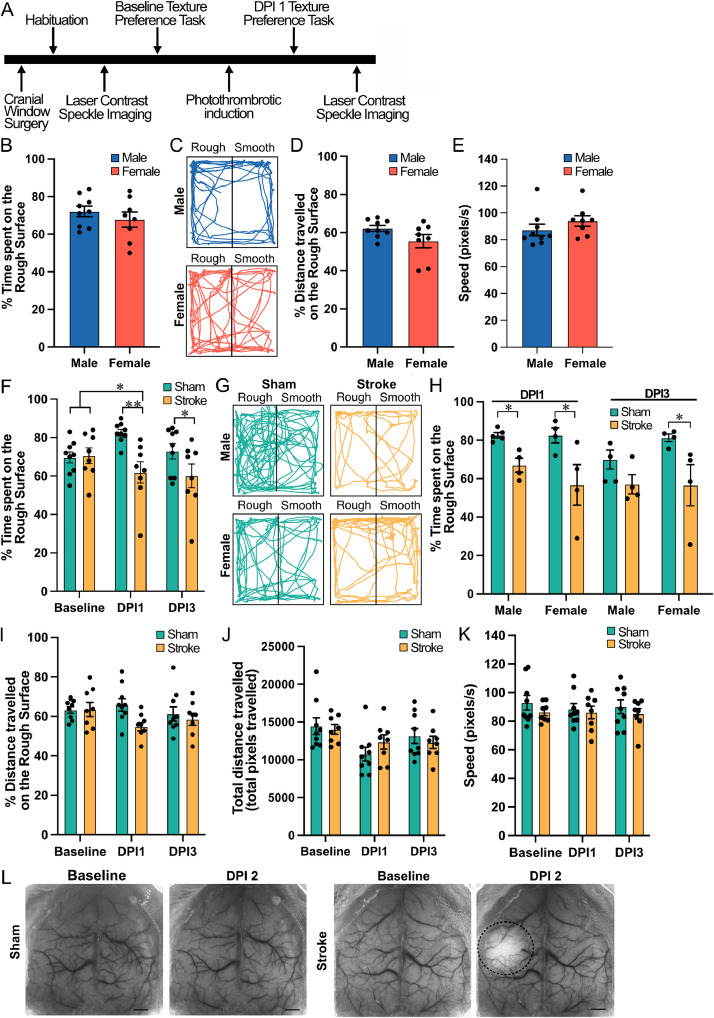
Experimental design and validation for the texture preference task. (A) Schematic of the experimental timeline. (B) Baseline percent time spent on the rough surface in naïve male and female mice. (C) Representative exploratory trajectories during the baseline texture preference task in males (top) and females (bottom), showing their preference for the rough surface during free exploration. (D) Baseline percent distance travelled on the rough surface in males and females, showing no sex difference in locomotor activity. (E) Baseline locomotor speed (pixels/s) in males and females, showing no sex difference in activity. (F) Percent time spent on the rough surface at baseline, DPI1 and DPI3 in sham and stroke groups. Stroke mice show a significant reduction in rough-surface preference at DPI1 and DPI3 relative to both baseline and sham controls. (G) Representative trajectories for sham and stroke mice at DPI1, separated by sex. (H) Sex-stratified analysis of percent time spent on the rough surface at DPI1 and DPI3, demonstrating a stroke-induced reduction in both males and females. (I) Percent distance travelled on the rough surface at baseline, DPI1 and DPI3 in sham and stroke groups. (J) Total distance travelled across the entire arena at baseline, DPI1 and DPI3 in sham and stroke groups. (K) Mean locomotor speed at baseline, DPI1 and DPI3 in sham and stroke groups, indicating preserved global activity. (L) Representative laser speckle contrast imaging (LSCI) at baseline and 2-days post-injury (DPI2) from sham and stroke mice, with the ischemic region delineated in the stroke condition (dashed outline). Sham (N = 5 Male; 4 Female); N = 8 stroke (N = 4 Male; N = 4 Female). Bars represent mean ± SEM with individual data points overlaid. Statistical significance: *p < 0.05, **p < 0.01, ***p < 0.001.

Most studies of tactile preference have used head-fixed paradigms with binary go/no-go discrimination tasks [[6], [7], [8]]. Although mechanistically informative, naturalistic spontaneous behaviours are overridden by the demand to produce a “correct” choice. Free moving assays offer a more direct view of innate tactile preference since sensory processing and attention occur in a naturalistic context. Recent work in rats has shown that rodents exhibit a robust preference for rough surfaces over smooth ones during free exploration [9]. Building on this, we sought to adapt and simplify the paradigm for mice, providing a freely moving open-field assay that requires minimal training and captures innate tactile-driven behavior. Fig. 1.

Knowing that stroke often disrupts tactile function, yet most behavioural assays emphasize gross motor outcomes, introducing a texture preference task offers a sensitive means to probe sensory-driven behaviour in this context [10,11]. Existing tasks such as adhesive removal or rotarod provide valuable information on motor coordination, but they do not directly capture spontaneous tactile preference [12,13].

Our motivation for providing this protocol is twofold: (i) to supply a reproducible, low-cost method for evaluating somatosensory preference in mice, and (ii) to demonstrate its utility in the context of cortical stroke, where sensory deficits can be subtle but clinically relevant. By combining this behavioral measure with laser speckle contrast imaging to confirm stroke induction, researchers can obtain a sensitive and complementary readout of somatosensory function alongside traditional sensorimotor tasks.

This protocol may be particularly useful for groups studying neurovascular injury, recovery after stroke, and interventions targeting sensory processing. It can also be applied more broadly to investigations of tactile perception and environmental preference in freely moving mice.

## Description of the protocol

**Surgical Implantation of Cortical Window and Head Fixation Bar**. Implant cranial windows in mice to enable laser speckle contrast imaging and photothrombosis induction [14]. Anesthetize mice with isoflurane (3 % in pure oxygen for induction, 1.5–2 % for maintenance after skin incision; Fresenius Kabi Canada Ltd). Maintain their body temperature at 37 °*C* ± 0.5 °C on a heating pad and monitor continuously with a rectal thermometer (Harvard apparatus). Provide analgesia subcutaneously (0.5 mg/ml in saline, 0.01 ml/g; Carprofen/Rimadyl, Best Buy Medical Supplies Inc.) and protect their eyes with lubricant (Systane). Shave the scalp, clean sequentially with alcohol and providone-iodine (TEVA Canada), and inject a local anaesthetic (0.2 ml, lidocaine) subcutaneously. Expose the skull and secure the surrounding skin with cyanoacrylate (Vetbond). Apply clear dental cement (C&B Metabond) to the skull and fix a glass coverslip in place to establish a transparent cranial window. Cement a titanium head bar (Labeo Technologies Inc.) posterior to the window to permit stable head fixation during imaging sessions and stroke induction. Postoperatively, inject 0.5 ml of subcutaneous saline and place mice in a warmed recovery cage until ambulatory (∼30 min). Monitor mice for 48 h before being returning them to their home cages. Administer carprofen (0.01 ml/g) daily for two days [15].

**Texture Preference Task.** Conduct behavioural testing in an open-field arena (45 × 45 × 40 cm) constructed with white opaque acrylic walls. Habituate mice to the arena for 5 min on the day prior to testing by placing the mice in the center of the arena and allowing free exploration. For baseline measures, divide the arena floor into two equal halves: one-half covered with smooth white board paper and the other covered with coarse sandpaper (P36 grit) to provide a tactile contrast. Perform experiments in the darkness under a red overhead light and record behaviour using an overhead camera (30 frames per s, Arducam). Run male mice before female mice to maintain consistency across cohorts and minimize olfactory confounds. On the test day, place each mouse individually in the center of the arena and allow free exploration for 3 min ensuring to counterbalance surface position. Clean the arena thoroughly with 70 % ethanol between trials to eliminate residual olfactory cues. Quantify texture preference manually by measuring the total time spent exploring each floor type. Exclude trials if mice remain immobile for >50 % of the session, if tracking data are incomplete due to technical errors, or if animals exhibit abnormal motor behaviour (e.g. circling or dragging) unrelated to the experimental manipulation. Repeat the task following stroke induction to assess whether somatosensory tactile deficits are present. The discrimination index was calculated by the amount of time spent on the rough surface divided by the total time exploring the arena. All calculations were performed for periods where the animal was moving or stationary for <5 s.

**Laser speckle contrast imaging (LSCI)**. Use speckle contrast imaging to visualize cerebral blood flow dynamics before and two days after stroke induction. Perform imaging with the LightTrack OiS200 system (Labeo Technologies Inc.) [16]. Illuminate the cortical surface with a 785 nm infrared laser (7.2 mW), and capture images for 30 s with a scientific CMOS camera (1024 × 1024 resolution over a 10 × 10 mm field of view) at 10 Hz and 10 ms exposure time [16]. Calculated speckle contrast values (*k* = standard deviation/mean intensity) in MATLAB (Mathworks) and generate contrast maps to verify the location and spatial extent of the induced strokes.

**Photothrombotic stroke induction**. Habituate mice to head fixation for two consecutive days prior to stroke induction. Induce photothrombosis in the motor/somatosensory cortex according to Allen Brain Atlas coordinates (−3 mm medial-lateral axis, 0.25 mm anterior-posterior axis), avoiding large vessels and sutures. Inject mice intraperitoneal with Rose Bengal (0.015 g/ml in saline; 10 ml/kg; Sigma Aldrich, 198,250–5 G). Allow circulation for 2 min before illuminating the targeted cortical region with a 532 nm green laser (11 mW, ∼1.1 mm beam diameter) via galvanometric mirrors in an optical imaging system (Labeo Technologies Inc.). Continue illumination for 13 min. The interaction of the laser light with Rose Bengal generated reactive oxygen species, resulting in localized thrombosis. For sham mice, the cortex was illuminated for the same duration of time, followed by an injection of Rose Bengal. Remove mice from head fixation following induction and monitored daily until euthanasia at 5 days post-injury.

**Statistical Analysis.** Mice were tracked with the open-source software SLEAP [17]. We labelled 160 frames across videos from different mice that consisted of a single point centred on the mouse body. We used the top-down pipeline from SLEAP, which first trains a centroid model to locate the animal, followed by a centred instances model to locate the tracking point. Using a GPU, the training took several hours. These models were then used to infer the mouse position on all frames from all videos at ∼60 frames per second. Predictions were exported as CSV and analysed in MATLAB (Mathworks). In most frames, the mouse was correctly tracked. Rarely, the mouse position was not found or confused with objects outside the arena. Wrong positions were removed, and all missing data points were linearly interpolated from neighbouring values. To smooth the x and y coordinates, we used a moving average with a window of 5 frames (∼167 ms). From these coordinates, we calculated the time spent on the rough and smooth surfaces, the distance travelled between consecutive frames and the instantaneous velocity. We segmented the mouse behaviour by thresholding the instantaneous velocity to identify static periods and retained those longer than 5 s. Then, all periods during which the mice were not identified as static were considered as moving. All measures were computed on the first 178 s from the start of the test and associated with the respective texture within the arena. The time spent within the rough surface quadrant was manually scored blind and we observed similar performance indexes from both the manual and automatic tracking (Supplemental Figure 1).

## Protocol validation

As depicted in Fig. 1A, the experimental timeline outlines the sequence of procedures used for our protocol validation of the texture preference task, including baseline testing, stroke induction and post-stroke behavioural assessment. To validate the automatic texture preference task, we first assessed baseline performance in sham-operated mice of both sexes (Fig. 1B-E) to determine whether sex differences were present for the preference of the rough or smooth surfaces. At baseline, male and female mice showed a robust preference for the rough surface, spending ∼65–75 % of the duration on the rough surface (Fig. 1B-C). A Welch’s *t*-test was performed using GraphPad Prism 10 showing no difference between sexes for the preference for the rough surface (*t*(12) = 0.8918, *p* = 0.39). This preference was evident despite free exploration of the arena (Fig. 1C), indicating that the task reliably captures an ethologically relevant tactile preference. No sex differences were detected in the distance travelled on the rough surface or for the average locomotor speed (Fig. 1D-E), demonstrating that baseline performance was not confounded by sex-specific differences in locomotor activity. Since no sex-dependent differences were observed, we combined sexes to investigate the effects of stroke.

We next evaluated the sensitivity of the protocol to detect stroke-related changes in texture preference (Fig. 1F-J). Using a repeated-measures ANOVAs, followed by Tukey’s multiple comparisons tests we compared to the time spent, distance travelled on the rough surface, total distance travelled in the arena as well as the mouse speed (Fig. 1F, H-K). Male and female mice were equally separated into baseline conditions and underwent either photothrombotic stroke or sham conditions, with laser contrast imaging used to confirm the cortical ischemic event (Fig. 1L). At baseline, both future sham and stroke groups preferred the rough surface, spending ∼70–75 % of trial time on the rough surface (Fig. 1F-G). Following photothrombotic induction, a significant reduction in rough-surface preference was observed at DPI1 and DPI3, with stroke mice spending less time on the rough surface compared with their own baseline performance and with sham-operated controls (Fig. 1F-G), a finding consistent with the presence of somatosensory deficits. This effect was supported by a significant main effect of condition (sham vs stroke: F(1,15) = 9.251, *p* = 0.0082) and a significant DPI × condition interaction (F(1.675, 25.13) = 4.628, *p* = 0.0245), with no main effect of DPI alone (F(1.675, 25.13) = 1.408, *p* = 0.2610), consistent with a stroke-specific disruption of tactile preference rather than a general time effect.

When stratified by sex, both male and female sham mice maintained a strong preference for the rough surface across sessions, whereas photothrombotic stroke produced a marked reduction in time spent on the rough surface in both sexes at DPI1 and DPI3 (Fig. 1H). At DPI1, this effect was driven by a significant main effect of condition (sham vs stroke: F(1,13) = 13.63, *p* = 0.0027), with no main effect of sex (F(1,13) = 0.8538, *p* = 0.3723) and no sex × condition interaction (F(1,13) = 0.8211, *p* = 0.3813). Similarly, at DPI3, stroke remained the primary determinant of reduced rough-surface preference (main effect of condition: F(1,13) = 8.687, *p* = 0.0113), with no main effect of sex (F(1,13) = 0.7214, *p* = 0.4110) and no sex × condition interaction (F(1,13) = 0.8606, *p* = 0.3705), indicating comparable somatosensory deficits in males and females (Fig. 1H).

This effect occurred in the absence of major changes in overall exploration since the percentage of distance travelled on the rough surface and the mean locomotor speed were comparable across sham and stroke conditions and remained stable between baseline, DPI1 and DPI3 (Fig. 1I-K). Specifically, this effect occurred in the absence of major changes in overall exploration, as the percentage of distance travelled on the rough surface (Fig. 1I) did not differ between sham and stroke groups (main effect of condition: F(1,15) = 2.801, *p* = 0.1149), across time (main effect of DPI: F(2,30) = 0.9453, *p* = 0.3998), or as a function of their interaction (DPI × condition: F(2,30) = 2.210, *p* = 0.1272). Similarly, total distance travelled in the arena (Fig. 1J) was comparable between sham and stroke mice (main effect of condition: F(1,15) = 0.0182, *p* = 0.8945) and showed no interaction with time (DPI × condition: F(1.894, 28.41) = 1.002, *p* = 0.3761), despite a modest main effect of DPI (F(1.894, 28.41) = 4.234, *p* = 0.0263). Mean locomotor speed (Fig. 1K) also remained stable across groups and time (main effects of condition: F(1,45) = 1.797, *p* = 0.1868; DPI: F(2,45) = 0.1804, *p* = 0.8356; DPI × condition: F(2,45) = 0.1312, *p* = 0.8774). These findings confirm that the stroke-related changes in rough-surface preference cannot be attributed to changes in locomotor activity.

Taken together, these findings demonstrate that the texture preference task is a reproducible and sensitive assay that captures a baseline preference for rough over smooth textures, is not biased by sex or gross locomotor differences, and is sufficiently sensitive to detect subtle stroke-induced changes in tactile exploration within 24–72 h of photothrombotic induction.

## Limitations

1. ***A restricted behavioural repertoire:*** The texture preference task provides a simple readout (time spent on each floor texture), which may not capture subtle sensorimotor deficits. While the task can reveal gross tactile deficits after stroke, it does not directly model higher-order sensory processing or complex functional impairments observed in humans. This design choice is consistent with widely used spontaneous preference paradigms, including novel object recognition and texture discrimination assays, which rely on short single-session testing to probe sensory-driven exploration under minimally trained conditions [18,19]. Accordingly, this protocol is optimized for acute sensitivity and high throughput rather than for assessing longitudinal stability of tactile preference. Future studies may incorporate extended habituation periods or repeated testing sessions across multiple days to evaluate the temporal stability of texture preference.
2. ***Presence of inflammation and pain at 1 day post-injury (DPI1):*** Behavioural testing at DPI1 occurs within the acute post-stroke phase and may be influenced by transient systemic and neurological factors, including inflammation, pain, edema, and sickness behaviour, which can affect exploratory behaviour. Although DPI1 was selected to capture the earliest detectable behavioural impact of injury, this timepoint does not isolate subacute or chronic somatosensory dysfunction. To mitigate this concern, we verified that global locomotor activity was preserved at DPI1, with no differences in velocity or total distance travelled between stroke and sham mice, supporting the feasibility of early testing in this paradigm.
3. ***Influence of locomotor activity:*** Reduced exploration due to post-stroke fatigue, anxiety or motor impairments may confound interpretation. Exclusion criteria of >50 % immobility reduces this bias; however, it may induce a limit on usable data. In our case, we did not need to exclude any mice due to changes in locomotor activity.
4. ***Testing duration***: The 3 min time trial was selected to maximize throughput but may not be sufficient to detect subtle preferences in some mice. Longer trials may increase sensitivity of the task. Nevertheless, exploratory behavior is usually pronounced at the onset of exposure to a novel environment but tends to decline as the animal habituates. Consequently, increasing the session duration is unlikely to result in a linear increase in the number of epochs.
5. ***Difference in surface colour:*** Although the task is performed in the dark under infrared illumination to minimize visual input, the rough surface is darker than the smooth surface, which may introduce a residual visual or contrast-related bias if the illumination condition were not controlled appropriately. In our experiment, we are sure of the experimental lighting conditions. We warn people to be careful of illumination conditions when using the task.

## Strengths and advantages

1. ***Simplicity and efficiency:*** The protocol does not rely on learning by the mouse, is easy to implement, requires minimal specialized equipment, and can be integrated into existing behavioural setups. Trials are short, allowing for high-throughput assessment of multiple animals per session.
2. ***Non-invasive, low stress, and repeatable:*** Since the task is based on spontaneous exploration, it can be performed repeatedly in the same mice, enabling within subject longitudinal assessment before and after experimental manipulations such as stroke. The short duration and habituation minimize possible confounding anxiety effects.
3. ***Direct measure of tactile preference:*** The contrasting floor textures provide a straightforward and quantifiable readout of somatosensory function that is independent from motor performance.
4. ***Comparison with existing validated texture-*based tests:** Several texture-based assays are available to probe somatosensory function in freely moving rodents, but most rely on trained discrimination rather than spontaneous preference. For example, Morita et al. trained water-motivated rats to choose between rough and fine sandpapers or grooved versus smooth panels in an operant chamber, enabling construction of high-resolution psychometric functions for whisker-based texture discrimination [5]. In mice, Wu and colleagues developed a whisker-dependent go/no-go texture task in which animals learn within a few days to discriminate textured panels using their mystacial vibrissae, requiring repeated handling and structured trials [18]. In parallel, cortical mechanisms underlying learned texture discrimination have been extensively characterized using operant whisker-based tasks; notably, O’Connor and colleagues demonstrated that precise neuronal encoding in barrel cortex is required for texture-guided decisions during goal-directed behavior [20]. More recently, textured Novel Object Recognition Tests (tNORT) have exploited rodents’ innate novelty preference by varying object surface texture and have been refined for repeated assessment of whisker sensitivity in disease models [4]. Compared with these paradigms, the present texture preference assay uses a simple two-zone rough-versus-smooth floor in an open field and quantifies spontaneous tactile preference via automated tracking of zone occupancy, without water or food restriction, explicit reinforcement, or multi-day training. This design preserves ethologically relevant, unreinforced exploration while remaining compatible with post-stroke or otherwise vulnerable mice testing, where motivation, learning rate, and repeated handling can confound performance in conventional discrimination tasks.

## Related to a research article in progress

The authors declare that they have no known competing financial interests or personal relationships that could have appeared to influence the work reported in this paper.

## Data availability

Example videos, SLEAP models, tracking data, and the MATLAB code used for the analysis are available on figshare with the identifier doi:10.6084/m9.figshare.30885707.

## CRediT authorship contribution statement

**Jessika Royea:** Conceptualization, Methodology, Investigation, Formal analysis, Supervision, Writing – original draft, Writing – review & editing. **Ismaël Djerourou:** Methodology, Investigation, Formal analysis, Writing – review & editing. **Behiye Sanliturk:** Methodology, Investigation, Writing – review & editing. **Catherine Albert:** Investigation, Writing – review & editing. **Matthieu P. Vanni:** Conceptualization, Methodology, Resources, Supervision, Funding acquisition, Writing – review & editing.

## Declaration of competing interest

The authors declare that they have no known competing financial interests or personal relationships that could have appeared to influence the work reported in this paper.

## Appendix Supplementary materials

Image, application 1
