# Supplementary material for: Texture preference task: a rapid and training-independent behavioural assay to evaluate somatosensory perception in freely moving mice
Source: MethodsX. 2026 Feb 21;16:103843. doi: 10.1016/j.mex.2026.103843 (PMC12955191; doi:10.1016/j.mex.2026.103843)
Supplement: Supplementary file 1 [file mmc1.docx]

**Supplementary Material**

**
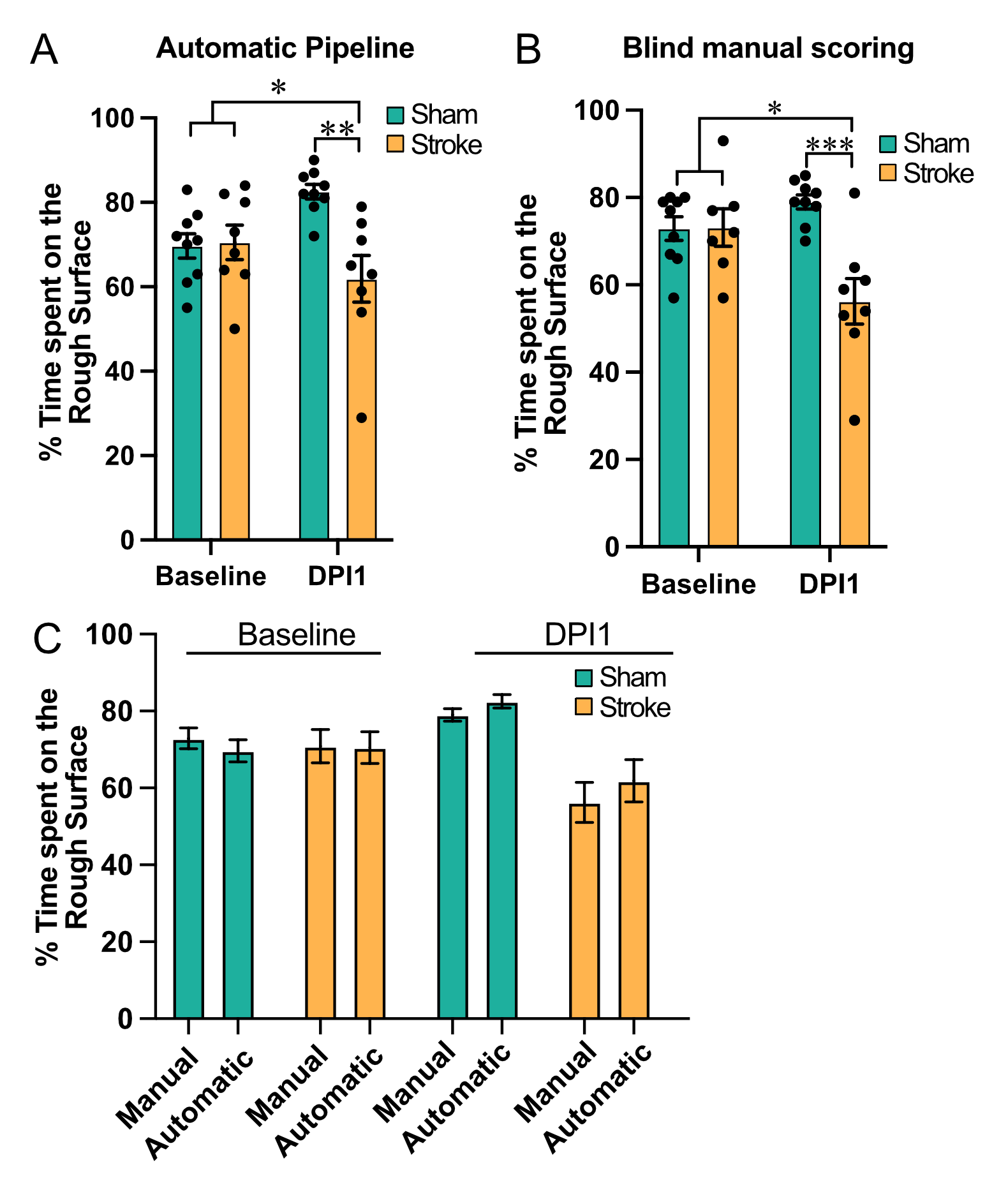
**

**Supplementary Figure 1. Validation of automated tracking for the texture preference task compared to manual scoring.** The percentage of time spent on the rough surface at baseline and 1 day post-injury (DPI1) quantified using an automated tracking program (A) and by blind manual scoring (B). Both methods revealed comparable behavioral patterns (C), with a significant shift in surface preference following injury. Individual data points represent single animals, bars show the mean ± SEM. Two way ANOVAs comparisons followed by Tukey’s multiple comparison’s tests were performed within each scoring method across time points and between textures (*p < 0.05, **p < 0.01, ***p < 0.001). A three way ANOVA comparing manual and automated scoring revealed no significant differences across conditions. These results demonstrate strong agreement between automated and manual scoring approaches, validating the automated pipeline for unbiased quantification of texture exploration behavior.
